# Culture and context shapes spiritual distress: A phenomenological study of spiritual distress in hospitalised people with advanced COPD in India

**DOI:** 10.1186/s12904-025-01896-y

**Published:** 2025-10-08

**Authors:** Barathi Bakthavatsalu, Catherine Walshe, Jane Simpson

**Affiliations:** 1 Division of Health Research, Faculty of Health and Medicine, Lancaster University, Sir John Fisher Drive, Lancaster, LA1 4AT UK; 2 International Observatory for End-of-Life Care, Division of Health Research, Faculty of Health and Medicine, Lancaster University, Sir John Fisher Drive, Lancaster, LA1 4AT UK

**Keywords:** Spiritual distress, Purposeless, Hospitalization, Advanced COPD, Qualitative study, Phenomenology

## Abstract

**Background:**

Spiritual distress appears common in people with advanced chronic obstructive pulmonary disease but is likely to be under-reported. Evidence indicates that spiritual distress becomes more intense during periods of hospitalisation when physical symptoms are exacerbated. Existing evidence on how such distress is experienced is mainly drawn from the Western context, which may not be appropriate to guide care for those from other cultures and contexts. The experience of spiritual distress in people with advanced chronic obstructive pulmonary disease in India is explored in this study.

**Methods:**

A descriptive, phenomenological approach was employed. People (*n* = 15) who were hospitalised with advanced chronic obstructive pulmonary disease were purposively sampled from an Indian tertiary care hospital in 2017. Unstructured interviews were conducted, audio-recorded, then transcribed. Analysis followed Giorgi’s method and themes related to spiritual distress were developed.

**Results:**

Three main themes were identified. (i) Purposeless life: repeated hospitalisation with acute breathlessness caused purposelessness but completing family responsibilities gave a sense of fulfilment. (ii) Despair and hope: extreme thoughts of ‘wishing to die but wanting to live’ were experienced alternately. (iii) Discontentment and death wish: Suffering caused feelings of abandonment by God, which triggered death wishes.

**Conclusions:**

This study has indicated that family and God were central to coping with spiritual distress during hospitalisation in Indian people with advanced chronic obstructive pulmonary disease. Identifying spiritual distress in its context and culture and the utilisation of appropriate spiritual support are important for palliative care professionals providing care to culturally diverse populations.

## Background

Chronic obstructive pulmonary disease (COPD) is the fourth major cause of mortality globally and by 2030 is likely to be the third most common [1]. The advanced stage of COPD is associated with increasing physical debility and high symptom burden affecting psychological and spiritual well-being of individuals [2, 3]. Although severe symptom burden and an unpredictable disease journey could contribute to high spiritual distress in people with advanced COPD, spiritual distress seems to be reported sparsely in people with advanced COPD [3–5]. It is important to study the experience of spiritual distress in people with advanced COPD.

Current evidence on the experience of spiritual distress for people in inpatient settings is mostly focused on individuals with cancer rather than for individuals with other chronic conditions such as advanced COPD [6, 7] Spiritual distress can be conceptualised and defined in different ways [8]. For this study, spiritual distress is defined as, *‘a state of suffering related to the impaired ability to experience meaning in life through connectedness with self*,* others*,* world or a Superior Being’* (8, p82).

The prevalence of spiritual distress in inpatient settings in a mixed population may range between 11 and 63% [6]. In contrast, in people with advanced COPD, spiritual distress can be higher in acute care settings (e.g. hospitals) due to unpredictable exacerbation of symptoms, than in non-acute care settings, such as outpatient and community settings [7]. However, spiritual distress in hospitalised people gets little attention due to healthcare staff’s bio-physical focus of the illness [6]. Hence, studying the experience of spiritual distress in hospitalised people with advanced COPD would enable healthcare professionals better understand, recognise and respond to this.

Currently, the experience of spiritual distress in hospitalised patients is reported mostly from Western perspectives [5, 6, 7, 9]. In the few studies which have compared a Western and Asian context, the importance of family is emphasised in Asian culture and autonomy in Western culture [10, 11]. For example, being with family was a means of connectedness in Koreans, in contrast to finding a spiritual connection through nature and being alone in Swedes [12]. Since these contextual and cultural factors play a major role in shaping the experience of spiritual distress, it is important to understand how spiritual distress is experienced in a non-Western culture and context [13]. This may help palliative care professionals provide culturally appropriate spiritual support to these populations. Consequently, this study aims to explore the experience of spiritual distress during hospitalisation in people with advanced COPD in the Indian context. This paper reports a further analysis of data collected as part of a study exploring the understanding of the experience of hospitalisation in people with advanced COPD in India [14].

## Methods

A qualitative, descriptive, phenomenological approach was employed [15]. This study was conducted in a tertiary care hospital in south India, in the pulmonary and geriatric in-patient wards, which were common settings for the care of people with advanced COPD. A purposive, homogenous sampling method was utilised [16]. Adult participants (age 18 and over) at an advanced stage of COPD, indicated by GOLD stage III and IV, and who were current in-patients were eligible for this study [2]. People with significant cognitive difficulties and communication challenges were excluded. Clinical staff from pulmonary and geriatric medicine wards identified potential participants from admission records. They explained the nature of the study and then gave the participation information sheet. Potential participants expressed their interest in participating in the study within three days of admission, either to clinical ward staff or the first author (BB). BB explained the voluntary nature of participation and ability to withdraw from the study. Written consent was taken (BB) prior to study participation. None of the participants were compensated monetarily. Some refreshments were provided after the interview as a token of appreciation for their time.

### Data collection

Individual, face-to-face, unstructured interviews were conducted in the Tamil language, on the in-patient wards. As most of the participants were physically frail, they were given the option of being accompanied by a family member. However, it was explained that the family member would not be allowed to take part in the interview. Open-ended questions were employed to explore topics related to spiritual distress. Data collection was conducted iteratively, in order to explore any new topics identified in the next interview. Although the phenomenon of spiritual distress was not the focus of the main study, this emerged as one of the elements of the phenomenon of hospitalisation in the first few interviews. Hence, this topic was added to the main interview guide/prompts to explore the phenomenon further in the subsequent interviews. The interview guide/prompts have been published previously [14]. Interviews were audio-recorded, transcribed into Tamil verbatim, then translated into English by the first author (BB). A bilingual expert facilitated back translation into Tamil to check the accuracy of the translation. NVivo 11 was used to store and manage the data. The first author (BB) coded the text and was verified by CW for consistency.

### Data analysis

Analysis followed Giorgi’s five-step, phenomenological method [15]. The text was read, then re-read to delineate ‘meaning units’ (codes) related to spiritual distress and hospitalisation, and these were coded. Data were coded inductively by BB and checked by CW for the consistency of coding. The meaning units were transformed into a third person format and to a formal language to facilitate the integration of similar descriptions from all transcripts. After eliminating irrelevant elements, main themes were developed. Themes related to spiritual distress were identified from the main themes of the experience of hospitalisation and were developed further.

### Ethics

Ethics approval was obtained from the authors’ host institution, Lancaster University Ethics Committee, (ID: FHMREC17006), institutional Ethics Committee, St. John’s Medical College, India (ID: IEC/169/2016), and from the Indian Council for Medical Research (ID: 5/8/4–31).

## Findings

Fifteen participants were approached and all agreed to participate (August to December 2017). The mean interview time was 26 min with a range of 20–30 min. The characteristics of participants are described in Table 1.

**Table 1:** Demographic characteristic of study participants

Baseline characteristics	Number/Mean
Gender	
Female	5
Male	10
Age (range)	61–83 years
Mean age	66.2
Religion	
Hindu	11
Christian	4
Time since diagnosis of COPD	Range 7–15 years
Route of admission to hospital	
Emergency department	10
Outpatient department	5
Length of hospital stay	Range 2–18 days
	Mean 4.73 days
Reason for hospitalisation	Acute breathlessness (*n* = 15)

Three themes related to spiritual distress were identified: purposeless life, despair and hope, and discontentment and death wish.

### A purposeless life

An emergency admission was often associated with physical dependency and anxiety and these factors were associated with feelings of purposelessness and questions around the meaning of life. Repeated emergency admissions caused a perception of imminent death which caused both loss of purpose in hospitalisation and in life more generally. A sense of worthlessness was perceived when individuals could not comprehend the meaning of life:

*“Why should I be alive? So far*,* I’ve worked hard; what am I going to earn hereafter? Am I going to buy some land or a car? Why should I be alive? [pause]…”* (P9).

Some individuals felt a purpose in life through holding a job and improving the quality of their living and, importantly, good physical strength. When none of these was met, this caused purposelessness.

Purposelessness also appeared to impact individuals’ faith in God. Some participants questioned God about their prolonged suffering; some felt abandoned by God, as they sensed that their illness became incurable. A Hindu participant expressed feelings of helplessness due to prolonged suffering, which limited participation in religious duties and rituals:

*“I want to go to the temple and worship to get some ‘punya’ [good deeds] …Oh Shiva [name of a Hindu god]. I am unable to do it now.”* (P11).

Religious duties were considered as good deeds by both Hindu and Christian people, which were perceived important to attain eternal life. Inability to perform religious duties meant both loss of fulfilment and purposelessness.

A perception of not being able to help others caused emptiness in life. However, family seemed to be the centre of having a purpose and providing some meaning in life. Completing family duties was regarded as having lived a meaningful life in the Indian context. Some participants felt completing family responsibilities helped them let go of life and prepared them to face what may lie ahead:

* “It’s enough; I’ve got my children married. I’ve seen all. [finished all duties as a family man].”* (P8).

Completing family responsibilities such as providing education for children and seeing them married gave a sense of purpose in life. Fulfilling these responsibilities was also considered as important societal roles and also related to individual religious beliefs.

### Despair and Hope

Prolonged suffering impacting physical, psychological and spiritual aspects was experienced due to repeated hospitalisation with breathlessness. Participants expressed a wish both to die and live during their extreme suffering while being hospitalised. Death wishes were felt during repeated acute breathlessness requiring hospitalisation, while thoughts of being around for family milestones, such as children’s weddings, helped change this to a wish to live. Inability to find meaning for individuals’ suffering caused despair and death wishes:

*“…am I going to work hard like carrying heavy loads? Or am I going to defend when strong men come to pick a fight with me? Why should I be alive? Whatever it is*,* let me end here [his life].”* (P9).

Nevertheless, religious beliefs and family were the two main sources of hope for individuals who felt a loss of meaning of suffering. Attributing suffering to past wrong deeds, i.e., ‘karma’, helped in coping and in finding a meaning for Hindu participants’ suffering:

*“…[sighs]… I don’t know what ‘karma’ I have done in the past [sins of the past life]*,* God has confined me [with illness].”* (P11).

Finding meaning in suffering also helped both Hindu and Christian individuals accept suffering as a punishment from God, which reduced the feeling of despair. Although Christian participants did not believe in karma, they related suffering as punishment for sin. As one Christian participant commented:

*“… If the mistake was unintentional I’ll get forgiveness. If the mistake was intentional then I will be punished for that.”* (P4).

This quote indicated that for this Christian individual believed both in forgiveness and punishment which could be the reason for sickness and suffering.

Life goals were usually centred on the family which seemed to be a source of hope for individuals. Living until completing these responsibilities encouraged hope and participants looked forward to enjoying the milestones of the family:

*“…let me see if it will get better and be alive for some time…. to support my son and to see my grandchildren.”* (P7).

Although the desire to live encouraged the hope for a longer future, uncertainty related to illness seemed to take away that hope, and participants were caught between a glimpse of hope and worries about the future. These thoughts appeared to alternate and reoccur, with some individuals feeling constantly being caught in contradicting cycles of despair and hope:

*“What is this illness and cough; I wish to do something to end my life [pause] let me see for another two or three years; I want to see my grandchildren; then*,* I will lay that idea back.”* (P9).

Family also facilitated coping with suffering and death wishes. Those who felt that they had completed their family responsibilities wanted to let go of life when faced with extreme difficulties in health. For some individuals letting go of life created a sense of helplessness and vulnerability, which caused death wishes:

*“…what can I do hereafter? That’s why; I don’t want to be alive. I want to die.”* (P2).

At these moments some people wondered about the purpose of hospitalisation and treatment, although they did appreciate the immediate symptom relief provided during hospitalisation. Loss of meaning for suffering caused death wishes, but finding meaning in life gave a desire to live longer and well until death.

### Discontentment and death wish

Constraints in participants’ relationships with God were perceived during extreme breathlessness. Physical discomforts and uncertainties associated with treatment triggered a perception of discontentment with God, leading to questioning God and His capability to cure their illness. Particularly, individuals who perceived that they had done good to others and followed their religious duties experienced a loss of connection with God more often than those who began to seek God after the diagnosis. These individuals felt abandoned by God during hospitalisation with extreme suffering which gave no indication of abating:

*“What is this God? Either take away [my] life or cure…do it at once; why God did this to me?”* (P4).

Questioning God reflected individuals’ discontentment with God due to prolonged illness and suffering. Prolonged suffering distanced people from God as some Hindu participants feared that reconciling with God was important to cleanse their past sins, ‘karma’, and to have eternal life.

Despite this ongoing suffering, for some individuals, becoming connected with God and others seemed to be important to cope with spiritual distress. Individuals restored this connection with God through performing religious rituals and by performing good deeds following hospitalisation. Performing religious activities along with their family and friends seemed to restore this connectedness:

*“…despite suffering like this*,* I am building a temple with the help of my friends…”* (P1).

For this Hindu participant, building a temple was perceived to restore hope and connection with God. Performing religious rituals such as ‘puja’ (worship) were also considered important for Hindu participants to connect with God.

Christian participants considered reconciliation with God and with others before death was important to connect with God:

*“I pray to God that if I have done any mistakes or sinned to forgive me. (pause) I want to make peace with everyone. I will forgive everyone who betrays me.” (P4)*.

#### Death wishes

Death wishes seemed to emerge when participants perceived emptiness of life, a loss of control over their illness and a disconnection from God. The first two were discussed in themes 1&2; here death wishes related to the loss of relationship with God is discussed.

Thoughts of death and dying concerns were related to individuals’ religious beliefs. Death wishes were also perceived when individuals felt a disconnection with God. Prolonged suffering caused individuals to question God’s ability to cure, which caused a sense of abandonment by God. This sense of abandonment triggered death wishes. Some individuals bargained with God, ‘either take away life or cure’. This individual expressed his wish to die explicitly; he was concerned that his progressive weakness and loss of productivity could burden his family members who took care of him:

*“I want to die [laughs loudly]…I am not useful for anybody [pause] for people who look after me also difficult…” (P8)* (Hindu participant).

Although both Hindu and Christian participants experienced fear of death during acute breathlessness, they all accepted death as part of life and that God controlled both one’s life and death. Despite repeated hospitalisation, none of the participants appeared to have discussed death and dying concerns with hospital staff during hospitalisation.

## Discussion

The experience of spiritual distress in people who were hospitalised with advanced COPD was explored in this study. Three key themes were identified: a purposeless life, despair and hope, and discontentment and death wish. Spiritual distress was mainly expressed in relation to loss of meaning of life, loss of meaning of suffering and loss of connectedness, which is discussed in relation to these three core dimensions identified in this study [8]. Individuals in this study identified culturally centred coping strategies,suchasfamilysupport,religiousbeliefsandrituals,whichwerehelpfultocombatspiritualdistress,whicharediscussed in this section.

### Loss of meaning in life

Loss of meaning is the core dimension of spiritual distress, identified across studies conducted in varied patient populations, cultures and contexts [4, 7, 13]. Meaning-making is important to gain a sense of control during illness [17, 18]. In this empirical study, the main source of finding meaning was centered on family and God/religion. Finding meaning in the past, present and future was important, and alwayscentered around family milestones such as children’s marriages. Treatment decisions often centered on family milestones, as the family provided overall care and co-ordination and financial support for treatment. A study conducted to understand the meaning of life between German and Indian palliative care patients reported that Indian participants prioritised spirituality and family in comparison to German participants, who relied on nature and their pets to find meaning of life [11].Few studies conducted in the Indian context reported family as one of the sources for spiritual support, which is in line with the finding of our study [19, 20].This indicates that varied, individual cultural factors, such as family values, could influence finding meaning in spiritually distressed people.

A sudden decline in health leading to hospitalisation prompted death wishes; however, thoughts of meeting family milestones changed death wishes and gave a purpose to life. This alternating desire for death and life therefore reflected participants’ culturally-derived coping strategies centered on family. Such ambivalent thoughts have been previously reported, but the reason for these thoughts was not reported [5, 21, 22]. Further, these ambivalent thoughts demonstrate that their coping was dynamic, which also shows the potential for therapeutic intervention.

### Loss of meaning of suffering

Loss of meaning for individuals’ suffering was another dimension of spiritual distress at the end of life [9, 23]. This study demonstrated that finding meaning for individuals’ suffering through religion facilitated the acceptance of suffering and death, as opposed to the desire for intensive treatment to prolong life in non-Asian cultures [24, 25]. Studies from Europe have shown that people with COPD look for options to extend their life because of the perception that living with COPD is a ‘way of life’ and seek self-management; this is in contrast to having faith in God in the Asian-Pacific culture [26, 27]. This viewpoint could influence how people in this study coped with spiritual distress from chronic illness, utilising belief in God and family as a primary source of support. For example, in the Swedish population, individually-focused strategies such as being in nature and enjoying recreation were useful for coping with spiritual distress [12]. However, performing religious rituals is helpful in the Indiancontext[20].Understandingthesenuancescouldhelpinformthespiritualsupportprovidedtoculturallydiverse populations.

### Loss of connectedness

Loss of connectedness is one of the indicators of spiritual distress [7, 28, 29]. There are differing viewpoints related to loss of connectedness depending on distinct cultural and religious factors [10, 12, 29]. In some cultural contexts, connectedness is predominantly related to self, finding oneself through recreation, companionship, nature etc. and spirituality is rarely utilised to restore connectedness [10, 23]. Family and God/religion have often been found helpful to restore connectedness both with self and others in the Indian culture [30–32]. This empirical study showed that performing religious activities with family and friends helped restore connectedness and was considered a way of strengthening relationships. Sense of abandonment was associated with loss of connection with God, which caused spiritual distress. Religious rituals and reciting prayers gave a sense of safety and connectedness with God, which is also reported in other Asian/Indian studies [19, 20, 33–35]. Family and God could both provide spiritual support to cope with loss of connectedness during hospitalisation.

### Spiritual distress at the end-of-life

Death and dying concerns are the main cause of spiritual distress in people hospitalised with advanced COPD [3, 5]. Despite experiencing fear of death during breathlessness, participants in this study were not hesitant to talk about death and dying concerns and their wish to have a peaceful death. In Indian culture, death is generally considered as part of life, viewed as a way to end suffering and to enter eternity [31]. Completion of family duties was viewed as preparation for death, which is also reported in other studies conducted in the Indian context [20]. Studies conducted to understand spiritual concerns/needs in India have reported that religious prayer and faith in God were important for spiritual well-being and acceptance of suffering at the end-of-life [10, 32, 33]. These culturally-influenced perceptions have implications for palliative care professionals in their role delivering appropriate spiritual support at the end-of-life.

### Strengths and limitations

This is the first study to explore the understanding of spiritual distress in hospitalised people with advanced COPD in India. Although this study reports spiritual distress in the Indian context, it has specific implications for Indians who are also living in the international setting. Limitations of the study include the relatively short duration of the interviews, as people were still in hospital and recovering from an acute illness episode. This may have meant that issues raised could not be explored in as much depth as desired. However, conducting interviews during the period of hospitalisation enabled the phenomenon of spiritual distress while in hospital to be captured contemporaneously and in context, rather than as reflections after the event. Since spiritual distress was not the main focus of this study, some of the elements of the experience would not have been captured or not completely developed. The experience of spiritual distress/struggle could be usefully explored in future studies [36].

### Recommendations for practice, policy and future research

Identifying spiritual strengths specific to an individual, their culture and religion could enable palliative care professionals to provide appropriate spiritual support [37]. Developing appropriate educational and training programmes for palliative care professionals to identify spiritual distress is necessary [38, 39]. Current evidence guides the assessment of spiritual care needs and support predominantly from a Western, Christian culture [6, 40]. Hence, developing spiritual support, such as identifying individuals’ personal spiritual strengths and speaking to a spiritual/religious counsellor, could be helpful. These spiritual support measures should be developed tailored to individual cultural and religious contexts and incorporated into palliative care programmes for people living with advanced COPD in India.

## Conclusion

Spiritual distress in hospitalised people with advanced COPD mainly emerged due to questions relating to the meaning of life and suffering, and questions about their connectedness with God. Family and God in this study were central to coping with spiritual distress during hospitalisation in the Indian context. Training palliative care professionals to identify spiritual distress and develop appropriatespiritualsupport,takingaccountofindividualculturalbeliefs,isimportant.Thiswouldenablehealthcare professionals to offer appropriate spiritual support to facilitate holistic care for culturally diverse populations with advanced COPD.

## Data Availability

Data are available on application to the corresponding author for approved research purposes. Competing interest: The authors declare that there is no competing interest.
